# Adiposity and Serum Selenium in U.S. Adults

**DOI:** 10.3390/nu10060727

**Published:** 2018-06-05

**Authors:** Qiuan Zhong, Ruoxi Lin, Qingjiao Nong

**Affiliations:** 1 Guangxi Medical University, School of Public Health, Department of Epidemiology, Nanning 530021, China; qjnong@gmail.com; 2 Guangxi Colleges and Universities Key Laboratory of Prevention and Control of Highly Prevalent Diseases, Guangxi Medical University School of Public Health, Nanning 530021, China; 3 Johns Hopkins Bloomberg School of Public Health, Departments of Epidemiology and Welch Center for Prevention Epidemiology and Clinical Research, Baltimore, MD 21205, USA; rulin@jhsph.edu

**Keywords:** adiposity, body mass index, percent body fat, waist circumference, selenium

## Abstract

Requirements for selenium and other antioxidant nutrients are increased in pro-oxidant and pro-inflammatory conditions such as excess adiposity. Data concerning the association of excess general and central adiposity with circulating selenium concentrations, however, are limited. We examined the cross-sectional associations of body mass index (BMI), percent body fat (%BF), and waist circumference (WC) with serum selenium concentrations in 6440 men and 6849 women aged ≥20 years who participated in the U.S. Third National Health and Nutrition Examination Survey. In multivariable analyses, the average difference (95% confidence interval (CI)) in serum selenium comparing the highest with the lowest quartiles of BMI was −4.0 (−5.5, −1.6) ng/mL in both men and women. These inverse associations were evident after further adjustment for WC. For %BF, the average differences (95% CI) in serum selenium between the highest and the lowest quartiles of %BF were −1.7 (−4.2, 0.7) ng/mL in men and −4.5 (−7.0, −1.9) ng/mL in women. The inverse association in women persisted after adjusting for WC. For WC, the average differences (95% CI) in serum selenium between the highest and the lowest quartiles were −1.9 (−3.8, −0.1) ng/mL in men and −3.9 (−5.8, −2.0) ng/mL in women. After further adjustment for BMI, the inverse association became positive in men and null in women. Our findings suggest that general and central adiposity have different associations with serum selenium levels and that these associations may depend on gender.

## 1. Introduction

Selenium is an essential trace element involved in the defense against oxidative stress as a key component of glutathione peroxidase (GPx) and other selenoenzymes. Excess adiposity, even at low levels of overweight and obesity, has been associated with increased oxidative stress [1,2,3,4,5] and with decreased activity of antioxidant enzymes including GPx [6]. Excess adiposity may thus represent a pro-oxidative and pro-inflammatory state that increases requirements for selenium and other antioxidants and may lead to long-term changes in selenium levels. In addition, a number of reports have shown independent associations between increasing serum selenium and the prevalence of a variety of cardiometabolic outcomes such as diabetes [7,8], dyslipidemia [9,10,11], and hypertension [12], all linked to excess adiposity.

Since excess adiposity may affect both selenium levels and cardiometabolic endpoints, it is important to understand the relationship between adiposity (both general and central) and serum selenium to interpret any apparent metabolic effects of selenium. Studies of the association between adiposity and selenium, however, have been scarce and are largely limited to body mass index (BMI). In the U.S. Third National Health and Nutrition Examination Survey (NHANES III) [13], overweight and obesity were associated with low serum selenium (defined as <100 ng/mL) in both premenopausal women and men < 65 years of age, but detailed dose–response relationships and the role of central obesity were not considered. In the French Supplémentation en Vitamines et Minéraux Antioxydants (SU.VI.MAX) study [14], obesity (BMI ≥ 30 kg/m^2^) was associated with decreased serum selenium levels, but only in women.

The objective of our analysis was thus to evaluate the association of adiposity with serum selenium taking into account measures of both general adiposity (BMI and percent body fat (%BF)) and central adiposity (waist circumference (WC)) in the general U.S. adult population, a selenium-replete population.

## 2. Materials and Methods

### 2.1. Study Population

NHANES III, a complex multistage survey, was aimed at obtaining a nationally representative sample of the non-institutionalized U.S. population from 1988 to 1994. Among 17,030 individuals aged 20 years and older who participated in the household interview and medical examinations, we excluded 61 participants with missing BMI values, 1248 with missing WC values, 1277 with missing bioelectrical impedance measurements, 847 with missing serum selenium levels, and 308 missing other covariates of interest. The final sample included 13,289 participants (6440 men and 6849 women). The Centers for Disease Control and Prevention (CDC) Institutional Review Board approved the NHANES III protocols and all participants provided written informed consent.

### 2.2. Body Mass Index, Percent Body Fat and Waist Circumference

Participants underwent a standardized home interview by trained interviewers and a standardized physical examination at the Mobile Examination Center [15,16]. Weight was measured with an electronic load-cell scale and recorded in kilograms to two decimal places while the participants were wearing only a paper examination gown with paper pants and foam slippers. Standing height was measured to the nearest 0.1 cm by using a stadiometer. BMI was calculated by dividing weight in kilograms by standing height in meters squared. WC was measured at the high point of the iliac crest by using a measuring tape in a standing position, and recorded to the nearest 0.1 cm.

Bioelectrical impedance analysis (BIA) was conducted as a single tetrapolar measurement of resistance and reactance at 50 kHz taken between the right wrist and ankle in the supine position using a Valhalla 1990B Bio-Resistance Body Composition Analyzer (Valhalla Scientific, San Diego, CA, USA) [17]. Fat-free mass (FFM) was estimated using a validated prediction equation [18,19]: Males: FFM = −10.678 + 0.262 weight + 0.652 height^2^/resistance + 0.015 resistance; Females: FFM = −9.529 + 0.168 weight + 0.696 height^2^/resistance + 0.016 resistance. Total body fat (TBF) was estimated as the difference between weight and FFM, and %BF was estimated as (TBF/weight) 100%.

### 2.3. Serum Selenium

During the examination, fasting whole blood was collected from study participants in containers previously screened for selenium contamination. After clotting, specimens were centrifuged, and serum was harvested, frozen at −20 °C, and shipped to the NHANES Laboratory, Nutritional Biochemistry Branch, Division of Environmental Health Laboratory Sciences (EHLS), National Center for Environmental Health (NCEH), CDC. Serum selenium was measured by Zeeman atomic absorption spectrometry [20,21]. The limit of detection was 8 ng/mL. All participants had serum selenium concentrations above the limit of detection. The interassay coefficients of variation for serum selenium ranged from 4.0 to 6.4% throughout the study period. Detailed descriptions of laboratory procedures and quality control methods for serum selenium measurements are available elsewhere [22].

### 2.4. Other Variables

Information on age, sex, race/ethnicity, education, menopausal status, smoking, physical activity, and antihypertensive medication was based on interview questionnaires. Leisure-time physical activity in the past month was ascertained during the home interview. Physical activities were coded and classified according to the rate of energy expenditure using a standardized scheme [23]. Sedentary lifestyle was defined as no moderate or vigorous physical activities performed in the past month. Never smokers were defined as participants who reported having smoked < 100 cigarettes during their entire lives. Current smokers were defined as participants who had smoked ≥ 100 cigarettes and reported that they were smoking now or had serum cotinine levels ≥ 10 ng/ml. Former smokers were defined as participants who had smoked ≥100 cigarettes but were not current smokers. Serum cotinine was measured by isotope-dilution high-performance liquid chromatography/atmospheric pressure chemical ionization tandem mass spectrometry. Blood pressure was the average of up to six measurements conducted during the in-home interview and at the mobile examination center according to the standard protocol recommended by the American Heart Association.

Serum C-reactive protein (CRP) was measured by latex-enhanced nephelometry (Behring Diagnostics Inc., Somerville, NJ, USA), which is a low-sensitivity assay, and grouped in three categories (<0.3 (limit of detection), ≥0.3 to <1, ≥1 mg/dL). Serum total cholesterol and triglycerides were measured enzymatically by using a Hitachi 704 Analyzer (Boehringer Mannheim Diagnostics, Indianapolis, IN, USA). High density lipoprotein (HDL) cholesterol was measured following the precipitation of other lipoproteins with a polyanion/divalent cation mixture. Plasma glucose was measured using a hexokinase enzymatic method. Diabetes was defined as a fasting glucose ≥6.99 mmol/L, a non-fasting glucose ≥11.1 mmol/L, or a self-report of a prior diagnosis of diabetes with concurrent use of oral hypoglycemic agents or insulin [22].

### 2.5. Statistical Analysis

We categorized BMI, %BF, and WC into sex-specific quartiles, and performed all analyses stratified by sex. Multivariable linear regression was used to calculate the difference in serum selenium comparing BMI, %BF, and WC quartiles to the first quartile. Model 1 was adjusted for age (continuous) and race/ethnicity (non-Hispanic white, non-Hispanic black, Mexican American, other). Model 2 was further adjusted for education (<12 years, ≥12 years), smoking (never, former, current), serum cotinine (continuous), sedentary lifestyle (yes, no), and postmenopausal status for women (yes, no). Model 3 was further adjusted for diabetes (yes, no), CRP (<0.3, ≥0.3 to <1, ≥1 mg/dL), systolic blood pressure (continuous), antihypertensive medication (yes, no), total cholesterol (continuous), triglycerides (continuous), and HDL-cholesterol (continuous). In addition, the findings for BMI and %BF quartiles were further adjusted for WC (Model 4), and the findings for WC quartiles were adjusted for BMI (Model 4a) or by %BF (Model 4b). Tests for linear trends were obtained by including the medians for each category of BMI, %BF, and WC as continuous variables in the regression models.

To evaluate more detailed dose–response relationships, we modeled BMI, %BF, and WC using restricted quadratic splines with knots at the 5th, 50th, and 95th percentiles to provide a smooth yet flexible description of the relationship of BMI, %BF, and WC with selenium. All analyses were performed in STATA (version 12, Stata Corp LP, College Station, TX, USA) using the “svy” commands to account for the complex study design and sampling weights of NHANES III.

## 3. Results

### 3.1. Characteristics of Study Population

The average BMI, %BF, and WC values of study participants were 26.6 kg/m^2^, 24.0%, and 95.4 cm, respectively, among men, and 26.4 kg/m^2^, 35.1%, and 88.5 cm, respectively, among women. The average concentrations of serum selenium were 127.2 ng/mL in men and 124.5 ng/mL in women. Generally, men had higher levels or percentages than women for current smokers, systolic blood pressure, serum cotinine, and serum triglycerides, but had lower levels or percentages for level of education, sedentary lifestyle, antihypertensive medication, serum C-reactive protein, serum total cholesterol, and serum HDL-cholesterol (Table 1).

### 3.2. Relationship between Body Mass Index and Serum Selenium

**Figure 1:**
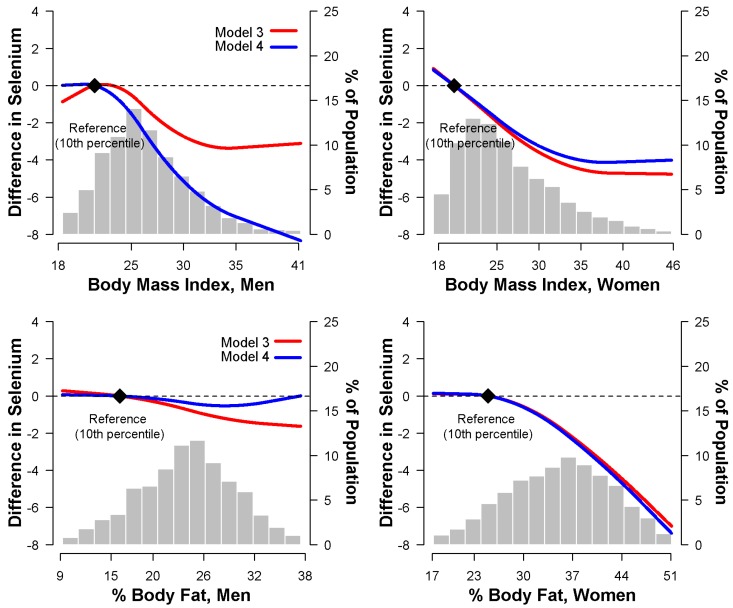
Multivariate-adjusted difference in serum selenium (ng/mL) associated with body mass index (kg/m^2^) and percent body fat (%), using restricted quadratic spline models. Model 3 (red line) was adjusted for age (continuous), race/ethnicity (non-Hispanic white, non-Hispanic black, Mexican American, other), education (<12 years, ≥12 years), postmenopausal status for women (yes, no), smoking (never, former, current), serum cotinine (continuous), sedentary lifestyle (yes, no), diabetes (yes, no), C-reactive protein (<0.3, ≥0.3 to <1, ≥1 mg/dL), systolic blood pressure (continuous), antihypertensive medication (yes, no), total cholesterol (continuous), triglycerides (continuous), and high density lipoprotein cholesterol (continuous). Model 4 (blue line) was further adjusted for waist circumference (continuous). The reference values (diamond dots) were set at the 10th percentile of the sample distributions of body mass index or percent body fat.

In multivariable adjusted models, higher BMI was associated with lower serum selenium concentrations in both men and women. The average difference in serum selenium between the highest and lowest quartile of BMI was −4.0 ng/mL (−5.5 to −1.6 ng/mL) in both men and women (Table 2, Model 3). After further adjustment for WC, the inverse association between BMI and selenium was stronger in men but somewhat weaker in women (Table 2, Model 4). Spline regression models confirmed that serum selenium concentrations decreased with increasing BMI, even after adjusting for WC (Figure 1).

### 3.3. Relationship between Percent Body Fat and Serum Selenium

With respect to %BF, multivariable adjusted models showed a strong inverse association with serum selenium in women, but only a weak and non-significant association in men. The average differences in serum selenium between the fourth and the first quartiles of %BF were −1.7 ng/mL (−4.2 to 0.7 ng/mL) in men, and −4.5 ng/mL (−7.0 to −1.9 ng/mL) in women (Table 3, Model 3). The inverse association in women was still evident after adjustment for WC (Table 3, Model 4) and in spline regression models (Figure 1).

### 3.4. Relationship between Waist Circumference and Serum Selenium

**Figure 2:**
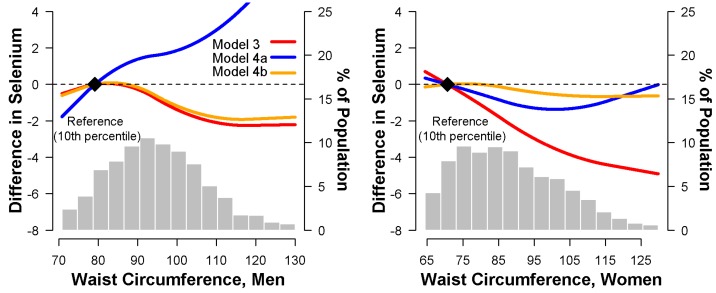
Multivariate-adjusted difference in serum selenium (ng/mL) associated with waist circumference (cm), using restricted quadratic spline models. Model 3 (red line) was adjusted for age (continuous), race/ethnicity (non-Hispanic white, non-Hispanic black, Mexican American, other), education (<12 years, ≥12 years), postmenopausal status for women (yes, no), smoking (never, former, current), serum cotinine (continuous), sedentary lifestyle (yes, no), diabetes (yes, no), C-reactive protein (<0.3, ≥0.3 to <1, ≥1 mg/dL), systolic blood pressure (continuous), antihypertensive medication (yes, no), total cholesterol (continuous), triglycerides (continuous), and high density lipoprotein cholesterol (continuous). Model 4a (blue line) was further adjusted for body mass index (continuous); and model 4b (orange line) was further adjusted for percent body fat (continuous) in addition to covariates in model 3. The reference values (diamond dots) were set at the 10th percentile of the sample distribution of waist circumference.

In multivariable adjusted models, higher WC was inversely associated with serum selenium in both men and women. The average differences in serum selenium between the fourth and the first quartiles of WC were −1.9 ng/mL (−3.8 to −0.1 ng/mL) in men, and −3.9 ng/mL (−5.8 to −2.0 ng/mL) in women, respectively (Table 4, Model 3). After adjustment for either BMI or %BF, the inverse association in both men and women became non-significant (Table 4, Models 4a,b). Spline regression models confirmed that serum selenium concentrations decreased with increasing WC (Figure 2, Model 3), but not after adjustment for BMI, which discrepantly presented a positive trend in men and a non-significant trend in women (Figure 2, Model 4a).

## 4. Discussion

In this nationally representative sample of the general U.S. population, BMI was inversely associated with serum selenium concentrations in both men and women, irrespective of WC, although the association was stronger in men, while %BF was inversely associated with serum selenium only in women. On the other hand, WC was positively associated with serum selenium in men within BMI, but the inverse association between WC and serum selenium in women was not persistent after considering BMI or %BF.

Previous studies of the association of BMI with serum selenium have been inconsistent. A report from 7423 women and 4915 men aged 35–60 years participating in the SU.VI.MAX study found lower selenium levels in obese than non-obese women though not in men [14]. A small study in Kuwait also found reduced serum selenium concentrations in patients with morbid obesity [24]. Also, the inverse relationship between serum selenium and obesity was observed even in children aged 6–17 years [25]. However, BMI was not associated with circulating selenium concentrations in 189 healthy British subjects [26], in a nationally representative sample of 1045 British adults [27], or in a cohort of 3387 Danish males aged 53–74 years [28]. In addition, in 245 adolescent girls living in rural Vietnam [29], BMI < 17 kg/m^2^ was found to be a risk factor for low serum selenium levels (<70 ng/mL), though this was likely a reflection of poor nutritional intake. Our analysis, conducted in a large representative sample of U.S. subjects, a selenium-replete population in contrast to the European populations mentioned above, showed that BMI was inversely associated with serum selenium concentration irrespective of WC. In our analyses, %BF was inversely associated with serum selenium only in women. The reasons for the differences in the associations of BMI and %BF with serum selenium by gender are unclear.

WC is a standard marker of abdominal and visceral fat [30], but to our knowledge, there have been no previous analyses on the association between WC and serum selenium in the general U.S. population. In our analyses that did not take BMI into account, WC was inversely associated with serum selenium in both men and women. In models that adjusted for BMI, however, the association between WC and serum selenium was positive in men and null in women. As there are large male–female differences in body composition, with women having higher levels of percent body fat but a less centrally located fat distribution [30], perhaps we should not be surprised by such gender differences. Interestingly, Charles et al. [31] showed that GPx activity was inversely associated with BMI but not WC in 110 police officers. Furthermore, the inverse association of GPx activity with BMI was significant only in men, but not WC regardless of men or women among the nationally representative 1045 British adults [27]. The with serum selenium concentrations needs to be confirmed in future studies and the underlying causes investigated in experimental studies.

The mechanisms underlying the inverse associations of BMI with serum selenium concentrations are not completely understood. Direct data on differences in selenium intake by BMI levels are unavailable and in any case, dietary questionnaires are an unreliable method of establishing selenium intake [32]. At first sight, a likely explanation is that a high BMI is associated with an inflammatory state which reduces circulating selenium [33,34]. This is at least partly because the expression of selenoprotein P, a major contributor to plasma selenium, is reduced in inflammation [35,36]. Circulating CRP concentration can be used as a measure of systemic inflammation. An elevation of high-sensitivity CRP of only 0.5 to 1 mg/dL was associated with a decrease in median plasma selenium of around 40% [34]. In our study, adjustment for CRP did not explain the association between serum selenium and adiposity markers, though it has to be noted that the CRP assay in NHANES III was a low sensitivity assay. Additionally, oxidative stress gradually increases during the development of overweight and obesity [2,4,5,37,38] and increasing adiposity could consume antioxidant selenoproteins in counteracting oxidative stress.

Previous studies have shown positive associations between serum selenium concentrations and a number of cardiometabolic risk factors, including diabetes, dyslipidemia, and hypertension [7,8,9,10,11,12], and some evidence suggests that these association may be modified by BMI [39]. The mechanisms underlying these associations are unknown, but our study suggests the possibility that even though serum selenium concentrations are inversely related to overall adiposity, for a given BMI level there may be a positive association between visceral adiposity and serum selenium levels, particularly in men. As with other biomarkers, serum selenium data thus need to be interpreted with care as they reflect not only selenium intake but also other underlying conditions that modify serum selenium concentrations.

Several limitations of this study need to be considered. Firstly, due to the cross-sectional design of the study, the direction of the associations cannot be established. Furthermore, the health implications of relatively small changes in selenium levels in a selenium replete population are uncertain. Secondly, although BMI, %BF and WC are widely used biomarkers of adiposity [40], they are not as reliable as more direct measures, such as dual-energy X-ray absorptiometry, in predicting systemic oxidative stress levels related to adiposity [41,42]. Thirdly, we only had measures of total serum selenium and no information on the serum selenoprotein concentration/activity or on other forms of selenium in serum. Future studies should evaluate different selenoproteins to better understand the association between selenium and cardiovascular risk factors. Finally, our analysis was based on a selenium-replete population, and our conclusions may not be generalized to other populations with marginal selenium intake.

## 5. Conclusions

BMI was inversely associated with serum selenium in both men and women, while %BF was inversely associated with serum selenium only in women. After BMI was taken into account, the associations between WC and serum selenium concentrations were positive in men and virtually null in women. General adiposity therefore appears to follow a different pattern of association with selenium compared to central adiposity. Future experimental and clinical studies will be needed to understand the link between body fat and selenium. Studies in populations with lower serum selenium concentrations are also needed to confirm these observations.

## Figures and Tables

**Table 1:** Baseline characteristics of the study population by gender †.

Characteristics	Overall (*n* = 13,289)	Men (*n* = 6440)	Women (*n* = 6849)	*p*
Age, year	44.2 (0.4)	43.0 (0.4)	45.3 (0.5)	0.75
White, %	77.2 (1.3)	77.3 (1.4)	77.2 (1.3)	0.88
Education (≥12 years), %	76.2 (1.1)	75.2 (1.2)	77.3 (1.1)	0.03
Current smoker, %	34.0 (0.9)	40.2 (1.0)	27.8 (0.9)	<0.001
Postmenopausal, %	—	—	39.6 (1.4)	
Sedentary lifestyle, %	34.8 (0.9)	27.0 (1.2)	42.4 (1.4)	<0.001
Systolic blood pressure, mmHg	122.2 (0.4)	124.3 (0.4)	120.0 (0.5)	< 0.001
Antihypertensive medication, %	11.6 (0.5)	9.7 (0.7)	13.5 (0.6)	< 0.001
Diabetes, %	5.3 (0.3)	5.6 (0.4)	4.9 (0.4)	0.18
Serum cotinine, ng/mL	78.5 (2.6)	96.1 (3.3)	61.1 (2.6)	< 0.001
C-reactive protein (≥1 mg/dL), %	7.0 (0.4)	4.4 (0.4)	9.6 (0.6)	< 0.001
Serum total cholesterol, mmol/L	5.3 (0.02)	5.2 (0.03)	5.3 (0.02)	0.001
Serum triglycerides, mmol/L	1.6 (0.02)	1.7 (0.03)	1.4 (0.02)	< 0.001
Serum HDL-cholesterol, mmol/L	1.3 (0.01)	1.2 (0.01)	1.4 (0.01)	< 0.001
Body mass index, kg/m^2^	26.5 (0.1)	26.6 (0.1)	26.4 (0.2)	< 0.001
Percent body fat, %	29.6 (0.2)	24.0 (0.2)	35.1 (0.3)	< 0.001
Waist circumference, cm	91.8 (0.3)	95.4 (0.3)	88.5 (0.4)	< 0.001
Serum selenium, ng/mL	125.8 (0.9)	127.2 (1.0)	124.5 (0.9)	< 0.001

† Values are means (SE) or percentage (SE) unless otherwise noted. HDL: High density lipoprotein.

**Table 2:** Difference (95% confidence interval (CI)) in serum selenium concentrations by quartiles of body mass index (BMI).

	Quartiles of BMI (kg/m^2^)				*p* for Trend
	Q1	Q2	Q3	Q4	
Men					
BMI, kg/m^2^	≤23.5	23.6–25.9	26.0–29.0	≥29.1	
Number (%)	1636 (25.4)	1466 (22.8)	1671 (26.0)	1667 (25.9)	
Serum selenium, ng/mL *	128.8	127.2	127.2	125.6	
Model 1 †	0.0 (reference)	−1.6 (−3.2, 0.2)	−0.8 (−3.2, 0.8)	−2.4 (−4.7, −0.8)	0.01
Model 2 ‡	0.0 (reference)	−1.6 (−3.2, −0.3)	−1.6 (−3.2, −0.1)	−3.2 (−4.7, −1.6)	0.003
Model 3 **	0.0 (reference)	−2.4 (−4.0, −0.8)	−2.4 (−4.0, −0.3)	−4.0 (−5.5, −1.6)	<0.001
Model 4 ††	0.0 (reference)	−3.2 (−4.7, −0.8)	−3.2 (−4.7, −0.8)	−5.5 (−8.7, −1.6)	0.005
Women					
BMI, kg/m^2^	≤21.9	22.0–25.1	25.2–29.9	≥30.0	
Number (%)	1317 (19.2)	1506 (22.0)	1985 (29.0)	2041 (29.8)	
Serum selenium, ng/mL *	125.6	125.6	124.0	123.2	
Model 1 †	0.0 (reference)	−0.2 (−1.6, 1.6)	−1.6 (−3.2, −0.1)	−2.4 (−4.0, −0.8)	0.006
Model 2 ‡	0.0 (reference)	−0.8 (−2.4, 1.6)	−1.6 (−3.2, −0.2)	−2.4 (−4.0, −0.8)	0.003
Model 3 **	0.0 (reference)	−1.6 (−3.2, 0.3)	−3.2 (−4.7, −1.6)	−4.0 (−5.5, −1.6)	<0.001
Model 4 ††	0.0 (reference)	−0.8 (−3.2, 0.8)	−2.4 (−4.0, −0.8)	−1.6 (−4.0, 0.2)	0.11

* Mean serum selenium levels. † Model 1: Adjusted for age (continuous) and race/ethnicity (non-Hispanic white, non-Hispanic black, Mexican American, other). ‡ Model 2: Further adjusted for education (<12 years, ≥12 years), postmenopausal status for women (yes, no), smoking (never, former, current), serum cotinine (continuous), and sedentary lifestyle (yes, no). ** Model 3: Further adjusted for diabetes (yes, no), C-reactive protein (<0.3, ≥0.3 to <1, ≥1 mg/dL), systolic blood pressure (continuous), antihypertensive medication (yes, no), total cholesterol (continuous), triglycerides (continuous), and HDL-cholesterol (continuous). †† Model 4: Further adjusted for waist circumference (continuous).

**Table 3:** Difference (95% CI) in serum selenium concentrations by quartile of percent body fat (%BF).

	Quartile of %BF				*p* for Trend
	Q1	Q2	Q3	Q4	
Men					
%BF	≤20.1	20.1–24.2	24.2–27.9	27.9–49.8	
Number (%)	1369 (21.3)	1510 (23.5)	1571 (24.4)	1990 (30.9)	
Serum selenium, ng/mL *	127.6	127.5	127.4	126.2	
Model 1 †	0.0 (reference)	−0.1 (−1.6, 1.4)	−0.2 (−2.5, 2.1)	−1.1 (−3.6, 1.4)	0.41
Model 2 ‡	0.0 (reference)	−0.5 (−2.0, 1.0)	−0.7 (−2.9, 1.5)	−1.3 (−3.8, 1.1)	0.31
Model 3 **	0.0 (reference)	−0.9 (−2.5, 0.7)	−1.3 (−3.5, 1.0)	−1.7 (−4.2, 0.7)	0.18
Model 4 ††	0.0 (reference)	−0.7 (−2.5, 1.0)	−0.9 (−3.8, 2.0)	−1.0 (−5.2, 3.1)	0.61
Women					
%BF	≤ 29.7	29.7–35.4	35.4–40.7	40.7–59.4	
Number (%)	1196 (17.5)	1633 (23.8)	1916 (28.0)	2104 (30.7)	
Serum selenium, ng/mL *	125.4	126.1	124.7	121.9	
Model 1 †	0.0 (reference)	1.0 (−0.6, 2.6)	−0.4 (−2.2, 1.4)	−2.8 (−4.7, −0.9)	0.002
Model 2 ‡	0.0 (reference)	0.7 (−0.9, 2.3)	−0.6 (−2.4, 1.2)	−3.1 (−5.0, −1.2)	0.001
Model 3 **	0.0 (reference)	−0.1 (−1.8, 1.6)	−2.2 (−4.4, 0.02)	−4.5 (−7.0, −1.9)	0.001
Model 4 ††	0.0 (reference)	−0.05 (−1.9, 1.8)	−2.0 (−4.7, 0.7)	−4.1 (−7.8, −0.4)	0.03

* Mean serum selenium levels. † Model 1: Adjusted for age (continuous) and race/ethnicity (non-Hispanic white, non-Hispanic black, Mexican American, other). ‡ Model 2: Further adjusted for education (<12 years, ≥12 years), postmenopausal status for women (yes, no), smoking (never, former, current), serum cotinine (continuous), and sedentary lifestyle (yes, no). ** Model 3: Further adjusted for diabetes (yes, no), C-reactive protein (<0.3, ≥0.3 to < 1, ≥1 mg/dL), systolic blood pressure (continuous), antihypertensive medication (yes, no), total cholesterol (continuous), triglycerides (continuous), and HDL-cholesterol (continuous). †† Model 4: Further adjusted for waist circumference (continuous).

**Table 4:** Difference (95% CI) in serum selenium concentrations by quartile of waist circumference (WC).

	Quartile of WC (cm)				*p* for Trend
	Q1	Q2	Q3	Q4	
Men					
WC, cm	≤86.2	86.3–94.4	94.5–102.9	103.0–168.8	
Number (%)	1616 (25.1)	1534 (23.8)	1602 (24.9)	1688 (26.2)	
Serum selenium, ng/mL *	127.3	128.1	126.8	126.5	
Model 1 †	0.0 (reference)	0.3 (−1.6, 2.3)	−1.0 (−3.2, 1.1)	−1.2 (−3.2, 0.7)	0.14
Model 2 ‡	0.0 (reference)	−0.1 (−2.0, 1.9)	−1.3 (−3.4, 0.8)	−1.5 (−3.4, 0.4)	0.08
Model 3 **	0.0 (reference)	−0.6 (−2.9, 1.6)	−1.7 (−4.2, 0.7)	−1.9 (−3.8, −0.1)	0.03
Model 4a ††	0.0 (reference)	0.1 (−2.0, 2.2)	−0.4 (−2.9, 2.2)	0.7 (−2.1, 3.4)	0.80
Model 4b ▲	0.0 (reference)	−0.5 (−3.3, 2.3)	−1.5 (−4.9, 1.8)	−1.6 (−5.1, 1.8)	0.29
Women					
WC, cm	≤76.6	76.8–86.4	86.5–98.4	98.6–157.8	
Number (%)	1269 (18.5)	1522 (22.2)	2019 (29.5)	2039 (29.8)	
Serum selenium, ng/mL *	125.5	125.2	124.2	123.2	
Model 1 †	0.0 (reference)	−0.4 (−2.2, 1.4)	−1.4 (−3.1, 0.4)	−2.3 (−4.0, −0.6)	0.005
Model 2 ‡	0.0 (reference)	−0.5 (−2.2, 1.3)	−1.3 (−3.1, 0.5)	−2.3 (−3.9, −0.6)	0.005
Model 3 **	0.0 (reference)	−1.0 (−2.8, 0.7)	−2.6 (−4.5, −0.6)	−3.9 (−5.8, −2.0)	<0.001
Model 4a ††	0.0 (reference)	−0.5 (−2.3, 1.3)	−1.5 (−3.7, 0.6)	−1.8 (−4.3, 0.7)	0.11
Model 4b ▲	0.0 (reference)	0.1 (−1.9, 2.1)	−0.6 (−3.3, 2.2)	−0.9 (−4.5, 2.7)	0.53

* Mean serum selenium levels. † Model 1: Adjusted for age (continuous) and race/ethnicity (non-Hispanic white, non-Hispanic black, Mexican American, other). ‡ Model 2: Further adjusted for education (<12 years, ≥12 years), postmenopausal status for women (yes, no), smoking (never, former, current), serum cotinine (continuous), and sedentary lifestyle (yes, no). ** Model 3: Further adjusted for diabetes (yes, no), C-reactive protein (<0.3, ≥0.3 to <1, ≥1 mg/dL), systolic blood pressure (continuous), antihypertensive medication (yes, no), total cholesterol (continuous), triglycerides (continuous), and HDL-cholesterol (continuous). †† Model 4a: Model 3 + body mass index (continuous). ▲ Model 4b: Model 3 + percent body fat (continuous).

## References

[1] Kelli H.M., Corrigan F.E., Heinl R.E., Dhindsa D.S., Hammadah M., Samman-Tahhan A., Sandesara P., O’Neal W.T., Al Mheid I., Ko Y.A. (2017). Relation of Changes in Body Fat Distribution to Oxidative Stress. Am. J. Cardiol..

[2] Fernandez-Sanchez A., Madrigal-Santillan E., Bautista M., Esquivel-Soto J., Morales-Gonzalez A., Esquivel-Chirino C., Durante-Montiel I., Sanchez-Rivera G., Valadez-Vega C., Morales-Gonzalez J.A. (2011). Inflammation, oxidative stress, and obesity. Int. J. Mol. Sci..

[3] Keaney J.F., Larson M.G., Vasan R.S., Wilson P.W., Lipinska I., Corey D., Massaro J.M., Sutherland P., Vita J.A., Benjamin E.J. (2003). Obesity and systemic oxidative stress: Clinical correlates of oxidative stress in the Framingham Study. Arterioscler. Thromb. Vasc. Biol..

[4] Galili O., Versari D., Sattler K.J., Olson M.L., Mannheim D., McConnell J.P., Chade A.R., Lerman L.O., Lerman A. (2007). Early experimental obesity is associated with coronary endothelial dysfunction and oxidative stress. Am. J. Physiol. Heart Circ. Physiol..

[5] Furukawa S., Fujita T., Shimabukuro M., Iwaki M., Yamada Y., Nakajima Y., Nakayama O., Makishima M., Matsuda M., Shimomura I. (2004). Increased oxidative stress in obesity and its impact on metabolic syndrome. J. Clin. Investig..

[6] Amirkhizi F., Siassi F., Djalali M., Shahraki S.H. (2014). Impaired enzymatic antioxidant defense in erythrocytes of women with general and abdominal obesity. Obes. Res. Clin. Pract..

[7] Wang X.L., Yang T.B., Wei J., Lei G.H., Zeng C. (2016). Association between serum selenium level and type 2 diabetes mellitus: A non-linear dose-response meta-analysis of observational studies. Nutr. J..

[8] Bleys J., Navas-Acien A., Guallar E. (2007). Serum selenium and diabetes in U.S. adults. Diabetes Care.

[9] Christensen K., Werner M., Malecki K. (2015). Serum selenium and lipid levels: Associations observed in the National Health and Nutrition Examination Survey (NHANES) 2011–2012. Environ. Res..

[10] Laclaustra M., Stranges S., Navas-Acien A., Ordovas J.M., Guallar E. (2010). Serum selenium and serum lipids in US adults: National Health and Nutrition Examination Survey (NHANES) 2003–2004. Atherosclerosis.

[11] Bleys J., Navas-Acien A., Stranges S., Menke A., Miller E.R., Guallar E. (2008). Serum selenium and serum lipids in US adults. Am. J. Clin. Nutr..

[12] Laclaustra M., Navas-Acien A., Stranges S., Ordovas J.M., Guallar E. (2009). Serum selenium concentrations and hypertension in the US Population. Circ. Cardiovasc. Qual. Outcomes.

[13] Kimmons J.E., Blanck H.M., Tohill B.C., Zhang J., Khan L.K. (2006). Associations between body mass index and the prevalence of low micronutrient levels among US adults. Med. Gen. Med..

[14] Arnaud J., Bertrais S., Roussel A.M., Arnault N., Ruffieux D., Favier A., Berthelin S., Estaquio C., Galan P., Czernichow S. (2006). Serum selenium determinants in French adults: The SU.VI.M.AX study. Br. J. Nutr..

[15] National Center for Health Statistics (1988). National Health and Nutrition Examination Survey Ш: Body Measurements (Anthropometry).

[16] Quinones J.L., Thapar U., Yu K., Fang Q., Sobol R.W., Demple B. (2015). Enzyme mechanism-based, oxidative DNA-protein cross-links formed with DNA polymerase beta in vivo. Proc. Natl. Acad. Sci. USA.

[17] Kuczmarski R.J. (1996). Bioelectrical impedance analysis measurements as part of a national nutrition survey. Am. J. Clin. Nutr..

[18] Chumlea W.C., Guo S.S., Kuczmarski R.J., Flegal K.M., Johnson C.L., Heymsfield S.B., Lukaski H.C., Friedl K., Hubbard V.S. (2002). Body composition estimates from NHANES III bioelectrical impedance data. Int. J. Obes. Relat. Metab. Disord..

[19] Sun S.S., Chumlea W.C., Heymsfield S.B., Lukaski H.C., Schoeller D., Friedl K., Kuczmarski R.J., Flegal K.M., Johnson C.L., Hubbard V.S. (2003). Development of bioelectrical impedance analysis prediction equations for body composition with the use of a multicomponent model for use in epidemiologic surveys. Am. J. Clin. Nutr..

[20] Lewis S.A., Hardison N.W., Veillon C. (1986). Comparison of isotope dilution mass spectrometry and graphite furnace atomic absorption spectrometry with Zeeman background correction for determination of plasma selenium. Anal. Chem..

[21] Paschal D.C., Kimberly M.M. (1986). Automated direct determination of selenium in serum by electrothermal atomic absorption spectroscopy. At. Spectrosc..

[22] Gunter E.W., Lewis B.G., Koncikowski S.M. (1996). Laboratory procedures used for the Third National Health and Nutrition Examination Survey.

[23] Ainsworth B.E., Haskell W.L., Leon A.S., Jacobs D.R., Montoye H.J., Sallis J.F., Paffenbarger R.S. (1993). Compendium of physical activities: Classification of energy costs of human physical activities. Med. Sci. Sports Exerc..

[24] Alasfar F., Ben-Nakhi M., Khoursheed M., Kehinde E.O., Alsaleh M. (2011). Selenium is significantly depleted among morbidly obese female patients seeking bariatric surgery. Obes. Surg..

[25] Blazewicz A., Klatka M., Astel A., Korona-Glowniak I., Dolliver W., Szwerc W., Kocjan R. (2015). Serum and urinary selenium levels in obese children: A cross-sectional study. J. Trace Elem. Med. Biol..

[26] Ghayour-Mobarhan M., Taylor A., New S.A., Lamb D.J., Ferns G.A. (2005). Determinants of serum copper, zinc and selenium in healthy subjects. Ann. Clin. Biochem..

[27] Spina A., Guallar E., Rayman M.P., Tigbe W., Kandala N.B., Stranges S. (2013). Anthropometric indices and selenium status in British adults: The U.K. National Diet and Nutrition Survey. Free. Radic. Biol. Med..

[28] Suadicani P., Hein H.O., Gyntelberg F. (1992). Serum selenium concentration and risk of ischaemic heart disease in a prospective cohort study of 3000 males. Atherosclerosis.

[29] Van Nhien N., Yabutani T., Khan N.C., Khanh le N.B., Ninh N.X., Chung le T.K., Motonaka J., Nakaya Y. (2009). Association of low serum selenium with anemia among adolescent girls living in rural Vietnam. Nutrition.

[30] Stevens J., Katz E.G., Huxley R.R. (2010). Associations between gender, age and waist circumference. Eur. J. Clin. Nutr..

[31] Charles L.E., Burchfiel C.M., Violanti J.M., Fekedulegn D., Slaven J.E., Browne R.W., Hartley T.A., Andrew M.E. (2008). Adiposity measures and oxidative stress among police officers. Obesity.

[32] Satia J.A., King I.B., Morris J.S., Stratton K., White E. (2006). Toenail and plasma levels as biomarkers of selenium exposure. Ann. Epidemiol..

[33] Nichol C., Herdman J., Sattar N., O’Dwyer P.J., St J.O.R.D., Littlejohn D., Fell G. (1998). Changes in the concentrations of plasma selenium and selenoproteins after minor elective surgery: Further evidence for a negative acute phase response?. Clin. Chem..

[34] Duncan A., Talwar D., McMillan D.C., Stefanowicz F., O’Reilly D.S. (2012). Quantitative data on the magnitude of the systemic inflammatory response and its effect on micronutrient status based on plasma measurements. Am. J. Clin. Nutr..

[35] Hesse-Bahr K., Dreher I., Kohrle J. (2000). The influence of the cytokines Il-1beta and INFgamma on the expression of selenoproteins in the human hepatocarcinoma cell line HepG2. Biofactors.

[36] Renko K., Hofmann P.J., Stoedter M., Hollenbach B., Behrends T., Kohrle J., Schweizer U., Schomburg L. (2009). Down-regulation of the hepatic selenoprotein biosynthesis machinery impairs selenium metabolism during the acute phase response in mice. FASEB J..

[37] Litwak S.A., Pang L., Galic S., Igoillo-Esteve M., Stanley W.J., Turatsinze J.V., Loh K., Thomas H.E., Sharma A., Trepo E. (2017). JNK Activation of BIM Promotes Hepatic Oxidative Stress, Steatosis, and Insulin Resistance in Obesity. Diabetes.

[38] Reho J.J., Rahmouni K. (2017). Oxidative and inflammatory signals in obesity-associated vascular abnormalities. Clin. Sci..

[39] Stranges S., Marshall J.R., Natarajan R., Donahue R.P., Trevisan M., Combs G.F., Cappuccio F.P., Ceriello A., Reid M.E. (2007). Effects of long-term selenium supplementation on the incidence of type 2 diabetes: A randomized trial. Ann. Intern. Med..

[40] Flegal K.M., Shepherd J.A., Looker A.C., Graubard B.I., Borrud L.G., Ogden C.L., Harris T.B., Everhart J.E., Schenker N. (2009). Comparisons of percentage body fat, body mass index, waist circumference, and waist-stature ratio in adults. Am. J. Clin. Nutr..

[41] Di Renzo L., Galvano F., Orlandi C., Bianchi A., Di Giacomo C., La Fauci L., Acquaviva R., De Lorenzo A. (2010). Oxidative stress in normal-weight obese syndrome. Obesity.

[42] Frossing S., Nylander M.C., Chabanova E., Kistorp C., Skouby S.O., Faber J. (2018). Quantification of visceral adipose tissue in polycystic ovary syndrome: Dual-energy X-ray absorptiometry versus magnetic resonance imaging. Acta Radiol..

